# Ultrasound Patterns in the First Trimester Diagnosis of Congenital Heart Disease

**DOI:** 10.3390/jcm10153206

**Published:** 2021-07-21

**Authors:** Catalin G. Herghelegiu, Anca Maria Panaitescu, Simona Duta, Ana Maria Vayna, Anca Marina Ciobanu, Cristian Bulescu, Raluca Gabriela Ioan, Adrian Neacsu, Nicolae Gica, Alina Veduta

**Affiliations:** 1 Department of Obstetrics and Gynecology, “Carol Davila” University of Medicine and Pharmacy, 050474 Bucharest, Romania; herghelegiu.cata@gmail.com (C.G.H.); anca.ciobanu@umfcd.ro (A.M.C.); cnr_cata@yahoo.com (R.G.I.); adrianneacsu2006@yahoo.com (A.N.); gica.nicolae@umfcd.ro (N.G.); 2 INSMC “Alessandrescu-Rusescu”, 020395 Bucharest, Romania; 3 “Filantropia” Hospital, 011171 Bucharest, Romania; duta_simona@yahoo.com (S.D.); anamariavayna@gmail.com (A.M.V.); alina.veduta@gmail.com (A.V.); 4 “Grigore Alexandrescu” Emergency Hospital for Children, 011743 Bucharest, Romania; cristianbulescu@gmail.com

**Keywords:** congenital heart disease, four-chamber view, three-vessel and trachea views, first trimester of pregnancy

## Abstract

Congenital heart disease (CHD) is the most common birth defect, with a reported prevalence of 5–12 per 1000 live births. Very recently, the American Institute of Ultrasound in Medicine published a guideline recommending the use of the four-chamber and the three-vessel and trachea views to screen for CHD in the first trimester of pregnancy. Our aim is to present abnormal image patterns that are seen in the four-chamber, three-vessel, and trachea views of the fetal heart in the first trimester and to describe their association with specific CHD types. We used a total of 29 cases of CHD from the archives of Filantropia Hospital and the Maternal and Child Health Institute (INSMC) fetal medicine units. We selected cases with a clear and well-documented diagnosis of the CHD type. We identified a series of repeating color doppler flow patterns seen in the four-chamber, three-vessel, and trachea views of the studied cases. Our observations could be developed into a diagnosis algorithm to orientate the examiner to the most likely type of CHD in individual cases.

## 1. Introduction

Congenital heart disease (CHD) is the most common birth defect, with a reported prevalence of 5–12 per 1000 live births [1,2].

There are many studies that show that the detection of major CHD in the first trimester of pregnancy is feasible [3,4,5,6,7,8,9,10,11]. Most of these studies acknowledge that correct and early diagnosis of the type of CHD still represents a great challenge [9,10,11,12,13]. Although the detection of CHD is the main objective of fetal ultrasound cardiac screening, correct identification of the CHD type is just as important, as different types of CHD can have very different prognoses and neonatal outcomes [1,14]. Timely and accurate prenatal diagnosis and the prediction of the probable postnatal outcome will allow parents to make informed choices in pregnancies affected by CHD.

Very recently, the AIUM (American Institute of Ultrasound in Medicine) published a guideline recommending the use of the four-chamber and the three-vessel and trachea views to screen for CHD in the first trimester of pregnancy [15]. Presently, there is limited information on the appearance of the four-chamber and the three-vessel and trachea views in cases of specific structural cardiac anomalies in the first trimester.

We retrospectively searched our database to identify the ultrasound presentation of various structural cardiac anomalies in the first trimester. This study was nested within the first trimester screening program in two public fetal medicine centers in Bucharest, Romania. Our aim is to present abnormal image patterns that are seen in the four-chamber and the three-vessel and trachea views of the fetal heart in the first trimester and to describe their association with specific CHD types. The recognition of such patterns might facilitate the correct diagnosis of the type of CHD.

## 2. Materials and Methods

The database we used is from two tertiary fetal medicine centers in Bucharest. We searched the archives of Filantropia Hospital and the Maternal and Child Health Institute (INSMC) fetal medicine units from January 2012 to June 2020 inclusively. There were 7812 cases (pregnant patients) and 7902 fetuses. It was a mixed population of screening and referred cases. The operators performing the first trimester examinations used a standardize protocol that we described in previous publications. This protocol includes the assessment of the four-chamber and three-vessel views of the fetal heart [10]. 

In our retrospective analysis, we included a total of 29 cases of CHD, with a clear and well-documented diagnosis of the CHD type and with at least reasonably good first trimester images of the heart. The 29 cases were diagnosed at the first trimester screening or were referred to the tertiary centers because of abnormal four-chamber and/or three-vessel and trachea views in the first trimester. The cases encompassed a large spectrum of structural heart defects (Table 1). The detailed/definitive diagnosis of the type of malformation was established by postnatal echocardiography, anatomopathological examination, or, at least, by second trimester echocardiography performed by a fetal cardiologist. 

We recovered and analyzed the four-chamber and the three-vessel and trachea view images of each case from the first trimester scans performed at one of the fetal medicine centers. The ultrasound images of the heart were obtained using doppler color flow mapping and high-frequency curvilinear abdominal transducers (4–9 Mhz, produced by GE - RM6C, RAB6-RS, RAB6-D, C2-9-RS) (GE Medical Systems, Zipf, Austria). We analyzed the patterns of the doppler color flow in relation to the type of CHD. We constructed a table that presents the images from the four-chamber, three-vessel, and trachea planes in a combined way that identified the doppler color flow patterns that might be specific for individual types of heart malformations. 

We used 20 matched non-CHD controls with known and normal outcomes to demonstrate the normal appearances upon doppler color flow. 

The patients involved gave specific consent for first trimester combined screening and general consent for the use of their clinical data for research. Ethical approval was not needed for this retrospective analysis of clinical data.

## 3. Results

We identified a series of repeating color doppler flow patterns seen in the four-chamber and the three-vessel and trachea views of the studied cases. The patterns we describe were based only on the color doppler inflow into the ventricles, as seen in the four-chamber view, as well as the ventricular outflow into the aorta and pulmonary artery, as seen in the three-vessel and trachea view. 

**Figure 1:**
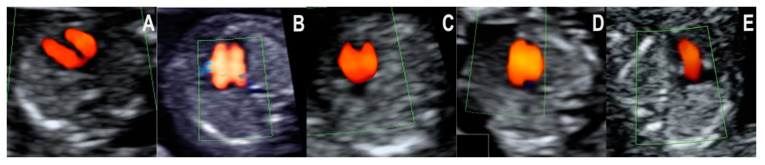
Color doppler flow patterns encountered in the four-chamber view. (**A**): Normal filling of both ventricles. (**B**): Filling of both ventricles, but the cardiac axis is abnormally shifted to the left. (**C**): Common inflow into the ventricles. (**D**): Filling of both ventricles, but the left ventricle appears smaller. (**E**): Filling of only one ventricle.

In the four-chamber view, the following five types of color doppler images were encountered (Figure 1A–E):

1A—Normal diastolic filling of both ventricles; the right and left inflows are clearly separated (by the interventricular septum), and the cardiac axis is normal (45–60 degrees).

1B—Normal diastolic filling of both ventricles; the right and left inflows are separated (by the interventricular septum), but the cardiac axis is abnormally rotated to the left.

1C—A common inflow into the ventricles through a common atrioventricular valve.

1D—Distinct filling of both ventricles, but the left ventricle appears smaller (shorter).

1E—Filling of only one ventricle, while the inflow for the other ventricle is completely absent.

**Figure 2:**
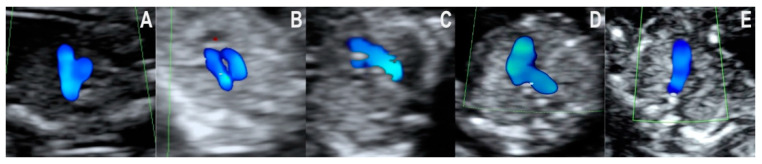
Color doppler flow patterns encountered in the three-vessel and trachea view. (**A**): Typical V sign with equally sized arms. (**B**): The vessels forming a U sign around the trachea. (**C**): V sign with a narrower arm. (**D**): One vessel with an oblique and curved course. (**E**): One vessel with a straight course.

In the three-vessel and trachea view, the following five types of color doppler images were seen (Figure 2A–E):

2A—The ductus arteriosus and the aorta form the typical V sign with equally sized arms.

2B—The ductus arteriosus and the aorta form equally sized arms, but the V sign cannot be seen.

2C—Both arms of the V sign are present, but one is narrower.

2D—Only one vessel can be observed with an oblique and curved course.

2E—Only one vessel can be observed with a straight, anterior-posterior course.

The diagnosis of the CHD type was confirmed by postnatal echocardiography (15 cases); first trimester anatomopathological examination (four cases); second trimester anatomopathological examination (six cases); postnatal anatomopathological examination (three cases); and second trimester echocardiography performed by fetal cardiologist (one case). The cases with a structurally normal heart all presented the same color flow pattern for the four-chamber, three-vessel, and trachea views.

The color doppler flow patterns and the distribution of cases in our analysis are shown in Table 2 and Table S1.

**Figure 3:**
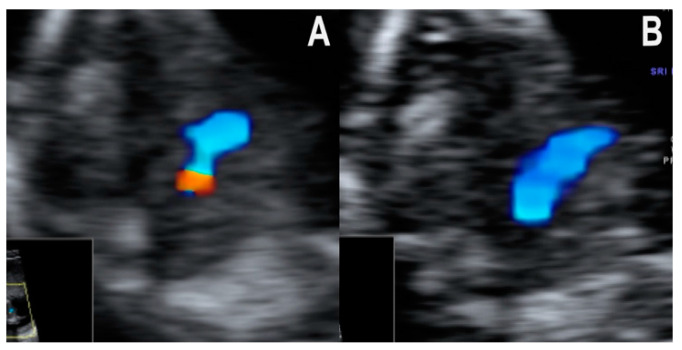
Color doppler flow patterns seen in the three-vessel and trachea view in the case of CAT. (**A**): Pulmonary arteries arising from the vessel. (**B**): The straight course of the vessel through the mediastinum.

We identified the following eight associations of the four-chamber, three-vessel, and trachea images (patterns):

1. Normal diastolic filling of both ventricles, with normal cardiac axis and two equally sized great vessels with normal antegrade flow, forming a V sign. This pattern was seen in all of the 20 cases with a structurally normal heart. The pattern’s negative predictive value for CHD that needs major surgical correction after birth is likely high, but little evidence is available to support this hypothesis. Isolated totally anomalous pulmonary veins return, and minor structural defects (e.g., ventricular septal defect, VSD), evolving lesions (e.g., mild valvular stenosis, tumors), or defects that are only postnatally diagnosed (e.g., atrial septal defect, ASD) might show this pattern in the first trimester.
2. Normal diastolic filling of both ventricles, with a normal cardiac axis and abnormal three-vessel and trachea view—the ductus arteriosus and the aorta form equally sized arms, but the V sign cannot be seen. The vessels are describing a U sign around the trachea. This pattern was seen in all three cases of RAA in our collection. In RAA, in the three-vessel and trachea view, the aorta is seen to the right of the trachea, and the ductus arteriosus encircles the trachea before joining the descending aorta.
3. Normal diastolic filling of both ventricles with normal cardiac axis and only one mediastinal vessel with curved course, at the level of the three-vessel and trachea view. This pattern was seen in the one case of TGA in our series. In the case of transposition, the great vessels are not in the same transverse plane exactly, so only one vessel is seen at the level of the three-vessel and trachea view, with a long trans-mediastinal of right to left course. TGA is a relatively rare lesion, therefore, data on the first trimester diagnosis of this disease are scarce in the literature.
4. Normal diastolic filling of both ventricles with an abnormal cardiac axis rotated to the left and one mediastinal vessel with curved course. All of the tetralogy of Fallot (TOF) and the double outlet right ventricle (DORV) cases in our collection respected this pattern. We found that the first trimester diagnosis was not difficult in the presence of the obviously abnormal curved, boomerang-like, trans-mediastinal vessel. On the other hand, we found it difficult to further differentiate between TOF and DORV (with no transposition of arteries) in early pregnancy. Based on the images analyzed, we think that DORV with transposed vessels might be distinguished from other lesions from the TOF spectrum on the basis of the longer trans-mediastinal course of the abnormal vessel.
5. Common inflow of both ventricles through a common atrioventricular valve and two equally sized great vessels with normal antegrade flow, forming a V sign. All of the atrioventricular septal defect (AVSD) cases in our series showed this pattern in the first trimester. AVSD with situs solitus is usually associated with genetic disorders, namely Down syndrome [16,17].
6. Filling of only one ventricle and one vessel with a straight course. The six hypoplastic left heart syndrome (HLHS) cases respected this pattern in the first trimester. The pattern was also seen in the one case of a univentricular heart from our collection. In the four-chamber view, only one ventricle (the right one in HLHS) has doppler inflow, while the other one is usually barely visible and has no doppler inflow. Correspondingly, in the three-vessel and trachea view, only one arterial vessel with doppler flow could be seen, namely the pulmonary artery with the ductus arteriosus in cases of a hypoplastic left heart. The prognosis of hypoplastic left heart syndrome, although not entirely predictable, is generally unfavorable, with no possibility of biventricular repair. The parents must be informed accordingly [1,18].
7. Distinct filling of both ventricles, but the left ventricle appears shorter (smaller), and two unequal vessels form the V sign (one vessel narrower). All the four cases of coarctation of the aorta (CoAo) seen in the first trimester showed this pattern. This type of CHD is a progressive disease. In our experience, the cases suspected in the first trimester are usually severe.
8. Normal diastolic filling of both ventricles with an abnormal cardiac axis and one mediastinal vessel with a straight course. We found this pattern in the one case of common arterial trunk from our collection. Filling of both ventricles was seen, but the cardiac axis was abnormally rotated to the left in the four-chamber view. At the level of the three-vessel and trachea view, one big central vessel that seemed to give rise to both pulmonary arteries and the aortic arch was seen (Figure 3A,B). Of note, the single vessel had a rather straight course. In cases of the more frequent and better-known conotruncal malformations, TOF and DORV, the dominant vessel is characteristically curved [8], but no clear data on CAT are available. In this case, the diagnosis was confirmed only by second trimester echocardiography performed by a fetal cardiology specialist.

## 4. Discussion

The table we constructed based on our observations of color doppler flow patterns of individual types of CHD can serve to orient the examiner to the most likely type of CHD. We chose to analyze images from two transverse planes: the four-chamber plane and the three-vessel and trachea plane, to comply with the recent AIUM recommendations [15]. These images offer valuable information about the four chambers of the heart, the two ventricles and two atria, the tricuspid and mitral valves, and the great arteries at the base of the heart, the aorta and pulmonary trunk [1,19,20,21].

An obvious limitation of our study is the small number of cases. Confirmation of the CHD type in diagnosis is inherently difficult in first trimester cases. Postnatal diagnosis, post-mortem examination, and even second trimester ultrasound examination are not always available. Pathologic examination of the first-trimester products of conception is not always possible [10,11,19]. 

There have been previous attempts to describe a simple standardized technique for the examination of the fetal heart in the first trimester of pregnancy [8,22,23]. In 2015, Wiechec et al. published similar observations on a similar number of CHD cases (35) from a seemingly selected population [8]. Unlike our table, that article presents less systematically the combinations of images in the four-chamber and the three-vessel and trachea planes that define overall patterns for the common structural heart anomalies. Although both our work and Wiechec’s article present descriptions with no quantified elements, the patterns described are recognizable, and could be useful for practitioners. 

Attempts to standardize and simplify the early examination of the fetal heart are all useful. While the technique used for the ultrasound examination of the fetal heart in the second trimester is clearly described and standardized [1,24,25,26], the technique for ultrasound examination of the fetal heart in the first trimester has been less defined [27]. Only recently, the AIUM issued a guideline recommending the use of the four-chamber and the three-vessel and trachea views to screen for CHD in the first trimester of pregnancy [15].

**Figure 4:**
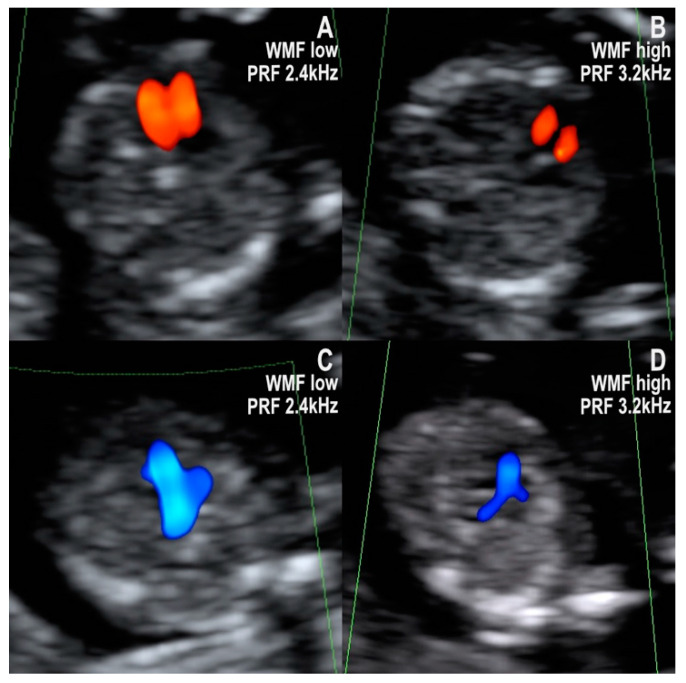
Color doppler flow patterns of the four-chamber, and three-vessel, and trachea views obtained using low and high PRF/WMF doppler settings in a normal 12–13-week fetus. (**A**): Low PRF/WMF doppler settings allow better visualization of the size and filling of the ventricles in the four-chamber view. (**B**): High PRF/WMF doppler settings are preferred to verify the distinct filling of the ventricles. (**C**): Low PRF/WMF doppler settings offer adequate visualization of the ductal and aortic arches. (**D**): High PRF/WMF doppler settings can impair the visualization of the aorta.

A key observation of using color doppler for fetal heart examination, especially in the first trimester, is that an adequate insonation angle and doppler settings—gain, pulse repetition frequency (PRF), and wall motion filter (WMF) are critical for obtaining useful images. In most cases, it is recommended to alternate low PRF/WMF and high PRF/WMF settings to better visualize the morphology of the heart/ventricles and the great vessels (Figure 4A–D). For example, for the four-chamber view, usually a high PRF/WMF is preferred to visualize the separation of the ventricles, but for the three-vessel and trachea view, usually a high PRF/WMF is preferred, as it permits to better visualize the arterial arches (Figure 4A–D). 

First trimester fetal ultrasound examination provides an opportunity for early diagnosis of congenital heart disease, with many potential benefits. The prenatal detection of certain congenital heart diseases, such as transposition of the great arteries, may result in appropriate anticipatory management and an improved outcome for the newborn [28].

The recognition of ultrasound patterns could facilitate the accurate diagnosis of the specific lesion in cases of CHD. Our observations could be developed into a diagnostic algorithm, but they need to be validated with bigger numbers and in prospective studies.

## Figures and Tables

**Table 1:** Types of CHD included in the study.

Type of Congenital Heart Disease	Number of Cases
Atrioventricular Septal Defect (AVSD)	6
Tetralogy of Fallot (TOF)	3
Double Outlet Right Ventricle (DORV)	4
Transposition of Great Arteries (TGA)	1
Unclassified Conotruncal Malformation/Common Arterial Trunk (CAT)	1
Hipoplastic Left Heart (HLHS)	6
Coarctation of the Aorta (CoAo)	4
Univentricular Heart	1
Right Aortic Arch (RAA)	3

**Table 2:** Color flow patterns seen in the four-chamber and the three-vessel and trachea views in normal and abnormal cases.

	**Four-Chamber** **View**	**Pattern 1** Normal diastolic filling of both ventricles Normal cardiac axis at 45–60°	**Pattern 2** Normal diastolic filling of both ventricles cardiac axis rotated to the left	**Pattern 3** Common inflow for both ventricles through a common atrioventricular valve	**Pattern 4** Distinct filling of both ventricles, but one of the ventricles, the left one, appears shorter	**Pattern 5** Filling of only one ventricle
**Three-Vessel** **and Trachea** **View**						
**Pattern 1** The ductus arteriosus and the aorta form the typical V sign with equally sized arms		Normal (20)		AVSD (6)		
**Pattern 2** The ductus arteriosus and the aorta form equally sized arms, but the V sign cannot be seen/a U sign is seen around the trachea		RAA (3)				
**Pattern 3** Both arms of the V sign are present, but one is narrower					CoAo (4)	
**Pattern 4** Only one vessel can be observed with a curved course		TGA (1)	Conotruncal DORV (4) TOF (3) CAT?			
**Pattern 5** Only one vessel can be observed with a straight course.			CAT (1)			HLHS (6) univentricular heart (1)

AVSD—atrioventricular septal defect; RAA—right aortic arch; CoAo—coarctation of the aorta; TGA—transposition of great arteries; DORV—double outlet right ventricle; TOF—tetralogy of Fallot; CAT—common arterial trunk; HLHS—hypoplastic left heart syndrome.

## Data Availability

The data presented in this study are available on request from the corresponding author.

## Supplementary Materials

The following are available online at https://www.mdpi.com/article/10.3390/jcm10153206/s1, Table S1: Color flow patterns seen in the four-chamber and the three-vessel and trachea views in normal and abnormal cases.

## References

[1] Donofrio M.T., Moon-Grady A.J., Hornberger L.K., Copel J.A., Sklansky M.S., Abuhamad A., Cuneo B.F., Huhta J.C., Jonas R.A., Krishnan A. (2014). Diagnosis and Treatment of Fetal Cardiac Disease. Circulation.

[2] Hoffman J.I.E., Kaplan S. (2002). The incidence of congenital heart disease. J. Am. Coll. Cardiol..

[3] Becker R., Wegner R.D. (2006). Detailed screening for fetal anomalies and cardiac defects at the 11–13-week scan. Ultrasound Obstet. Gynecol..

[4] Rasiah S.V., Publicover M., Ewer A.K., Khan K.S., Kilby M.D., Zamora J. (2006). A systematic review of the accuracy of first-trimester ultrasound examination for detecting major congenital heart disease. Ultrasound Obstet. Gynecol..

[5] Smrcek J.M., Berg C., Geipel A., Fimmers R., Axt-Fliedner R., Diedrich K., Gembruch U. (2006). Detection rate of early fetal echocardiography and in utero development of congenital heart defects. J. Ultrasound Med..

[6] Persico N., Moratalla J., Lombardi C.M., Zidere V., Allan L., Nicolaides K.H. (2011). Fetal echocardiography at 11-13 weeks by transabdominal high-frequency ultrasound. Ultrasound Obstet. Gynecol..

[7] Iliescu D., Tudorache S., Comanescu A., Antsaklis P., Cotarcea S., Novac L., Cernea N. (2013). Improved detection rate of structural abnormalities in the first trimester using an extended examination protocol. Ultrasound Obstet. Gynecol..

[8] Wiechec M., Knafel A., Nocun A. (2015). Prenatal Detection of Congenital Heart Defects at the 11- to 13-Week Scan Using a Simple Color Doppler Protocol Including the 4-Chamber and 3-Vessel and Trachea Views. J. Ultrasound Med..

[9] de Robertis V., Rembouskos G., Fanelli T., Volpe G., Muto B., Volpe P. (2017). The three-vessel and trachea view (3VTV) in the first trimester of pregnancy: An additional tool in screening for congenital heart defects (CHD) in an unselected population. Prenat. Diagn..

[10] Vayna A.M., Veduta A., Duta S., Panaitescu A.M., Stoica S., Buinoiu N., Nedelea F., Peltecu G. (2018). Diagnosis of fetal structural anomalies at 11 to 14 weeks. J. Ultrasound Med..

[11] Duta S., Veduta A., Vayna A.M., Panaitescu A., Nedelea F., Peltecu G. (2019). The outcome of structural heart defects diagnosed in the first trimester of pregnancy. J. Matern. Fetal Neonatal Med..

[12] Kashyap N., Pradhan M., Singh N., Yadav S. (2015). Early Detection of Fetal Malformation, a Long Distance Yet to Cover! Present Status and Potential of First Trimester Ultrasonography in Detection of Fetal Congenital Malformation in a Developing Country: Experience at a Tertiary Care Centre in India. J. Pregnancy.

[13] Herghelegiu C.G., Duta S.F., Neacsu A., Suciu N., Veduta A. (2020). Operator experience impact on the evaluation of still images of a first trimester cardiac assessment protocol. J. Matern. Fetal Neonatal Med..

[14] Yeu B.K., Chalmers R., Shekleton P., Grimwade J., Menahem S. (2008). Fetal Cardiac Diagnosis and Its Influence on the Pregnancy and Newborn—A Tertiary Centre Experience. Fetal Diagn. Ther..

[15] (2021). AIUM Practice Parameter for the Performance of Detailed Diagnostic Obstetric Ultrasound Examinations Between 12 Weeks 0 Days and 13 Weeks 6 Days. J. Ultrasound Med..

[16] Syngelaki A., Chelemen T., Dagklis T., Allan L., Nicolaides K.H. (2011). Challenges in the diagnosis of fetal non-chromosomal abnormalities at 11-13 week. Prenat. Diagn..

[17] Veduta A., Vayna A.M., Duta S., Panaitescu A., Popescu F., Bari M., Peltecu G., Nedelea F. (2018). The first trimester combined test for aneuploidies–a single center experience. J. Matern. Fetal Neonatal Med..

[18] Perde F., Herghelegiu C.G., Iosifescu A.G., Crîngu I., Luca L., Dragu M. (2018). Pulmonary artery aneurysm in a marfanoid adult patient with unoperated functional single ventricle and levo-transposition of the great arteries. Rom. J. Leg. Med..

[19] Herghelegiu C.G., Neacsu A., Chircurescu R., Herghelegiu D., Voinea S.C., Diaconu C., Stiru O., Savu C., Filipescu A., Balescu I. (2021). Pathological examination of the late embryonic heart using the same 4-chamber and 3-vessel planes used in fetal echocardiography. In Vivo.

[20] Yoo S.J., Lee Y.H., Cho K.S., Kim D.Y. (1999). Sequential segmental approach to fetal congenital heart disease. Cardiol. Young.

[21] Yoo S.J., Lee Y.-H., Kim E.S., Ryu H.M., Kim M.Y., Choi H.-K., Cho K.S., Kim A. (1997). Three-vessel view of the fetal upper mediastinum: An easy means of detecting abnormalities of the ventricular outflow tracts and great arteries during obstetric screening. Ultrasound Obstet. Gynecol..

[22] Orlandi E., Rossi C., Perino A., Musicò G., Orlandi F. (2014). Simplified first-trimester fetal cardiac screening (four chamber view and ventricular outflow tracts) in a low-risk population. Prenat. Diagn..

[23] Quarello E., Lafouge A., Fries N., Salomon L.J. (2017). Basic heart examination: Feasibility study of first-trimester systematic simplified fetal echocardiography. Ultrasound Obstet. Gynecol..

[24] Yagel S., Cohen S.M., Achiron R. (2001). Examination of the fetal heart by five short-axis views: A proposed screening method for comprehensive cardiac evaluation. Ultrasound Obstet. Gynecol..

[25] Salomon L.J., Alfirevic Z., Berghella V., Bilardo C., Hernandez-Andrade E., Johnsen S.L., Kalache K., Leung K.-Y., Malinger G., Munoz H. (2010). Practice guidelines for performance of the routine mid-trimester fetal ultrasound scan. Ultrasound Obstet. Gynecol..

[26] Crino J., Finberg H.J., Frieden F., Kuller J., Odibo A., Robichaux A., Bohm-Velez M., Pretorius D.H., Sheth S., Angtuaco T.L. (2013). AIUM Practice Guideline for the Performance of Obstetric Ultrasound Examinations. J. Ultrasound Med..

[27] Carvalho J.S., Allan L.D., Chaoui R., Copel J.A., DeVore G.R., Hecher K., Lee W., Munoz H., Paladini D., Tutschek B. (2013). ISUOG Practice Guidelines (updated): Sonographic screening examination of the fetal heart. Ultrasound Obstet. Gynecol..

[28] Dastgiri S., Gilmour W.H., Stone D.H. (2003). Survival of children born with congenital anomalies. Arch. Dis. Child..

