# Supplementary material for: Ultrasound Patterns in the First Trimester Diagnosis of Congenital Heart Disease
Source: J Clin Med. 2021 Jul 21;10(15):3206. doi: 10.3390/jcm10153206 (PMC8347903; doi:10.3390/jcm10153206)
Supplement: Supplementary file 1 [file jcm-10-03206-s001.zip › jcm-1289855-supplementary.pdf]

Table S1–Colour Flow Patterns seen on the 4-chamber and 3-vessel and trachea views in normal and abnormal cases.

**4-chamber view** / **3-vessel and trachea view**	**Pattern 1** - nonturbulent filling of both ventricles normal cardiac axis 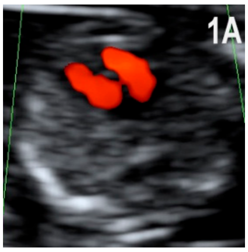	**Pattern 2** - nonturbulent filling of both ventricles cardiac axis shifted to the left 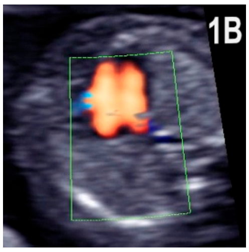	**Pattern 3** - common inflow for both ventricles 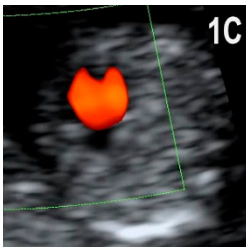	**Pattern 4** - one of the ventricles, the left one, appears shorter 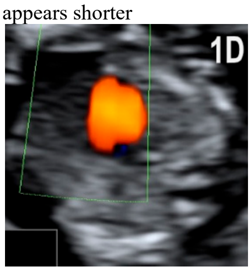	**Pattern 5** - filling of only one ventricle 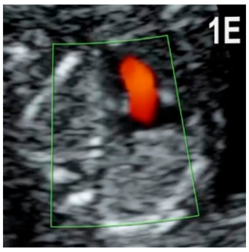
**Pattern 1** - the ductus arteriosus and the aorta form the typical V sign 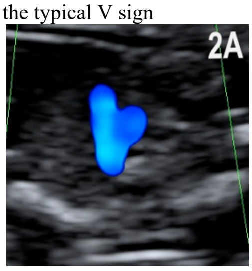	**Normal heart**		**AVSD**		
**Pattern 2** - the ductus arteriosus and the aorta form a U sign around the trachea 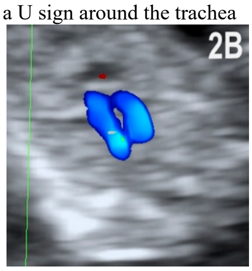	**RAA**				
**Pattern 3** - both arms of the V sign are present, but one is narrower 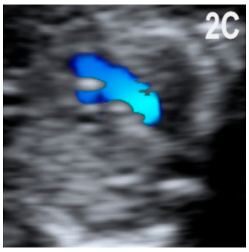				**CoAo**	
**Pattern 4** - only one vessel can be observed with a curved course 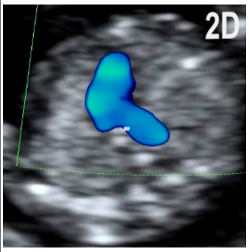	**TGA**	**Conotruncal malformation**<br>**DORV**<br>**TOF**<br>**CAT?**			
**Pattern 5** - only one vessel can be observed with a straight course. 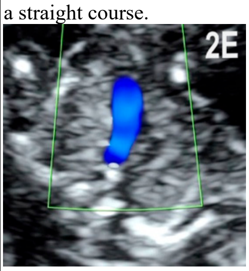		**CAT**			**HLHS**<br>**univentricular heart**

*Authors: Catalin G Herghelegiu, Anca Panaitescu, Alina Veduta. AVSD – atrioventricular septal defect; RAA – right aortic arch; CoAo – coarctation of the Aorta; TGA – transposition of great arteries; DORV – double outlet right ventricle; TOF – tetralogy of Fallot; CAT – common arterial trunk; HLHS – hypoplastic left heart syndrome
